# Point-of-care ultrasound-guided resuscitation and transport of an extremely premature infant in a pre-hospital setting: a case report

**DOI:** 10.3389/fped.2025.1709299

**Published:** 2026-01-20

**Authors:** Deng Bi-Ying, Li Jin-Feng, Chen Qin, Li Ning, He Xiao-Guang

**Affiliations:** 1 Neonatology Department, Dongguan Children’s Hospital Affiliated to Guangdong Medical University, Dongguan, Guangdong, China; 2 Key Laboratory of Neonatal Major Diseases, Dongguan Children’s Hospital Affiliated to Guangdong Medical University, Dongguan, Guangdong, China

**Keywords:** extremely premature infants, point-of-care ultrasound, pre-hospital setting, resuscitation, transportation

## Abstract

A male infant born vaginally with clear amniotic fluid at a gestational age of 24 weeks had Apgar scores of 6 at 1 and 5 min and 7 at 10 min and a birth weight of 600 g. After receiving pulmonary surfactant therapy administered through an endotracheal tube at the local hospital, he continued to exhibit severe respiratory distress and hypoxemia; moreover, obtaining peripheral venous access remained difficult despite mechanical ventilation. Consequently, the neonatal transport team from our hospital was called to assist with treatment and transfer. Upon arrival, the transport team used point-of-care critical ultrasound for dynamic assessment and obtained the following findings: (1) the lung ultrasound assessments excluded pneumothorax and helped optimize the ventilator parameters to achieve patient–ventilator synchrony; (2) endotracheal tube placement was confirmed; (3) cranial ultrasound was performed to screen for intracranial hemorrhage; and (4) ultrasound-guided umbilical arterial and venous catheterization was successfully performed to establish vascular access. Under mechanical ventilation support and continuous monitoring, the infant was successfully transported to our neonatal intensive care unit (NICU), requiring no repeat invasive procedures upon admission and maintaining a stable condition throughout transport. This case demonstrates the effectiveness of point-of-care critical ultrasound for real-time guidance during the resuscitation and transport of extremely preterm infants. By enabling multi-system evaluation that included lung, airway, vascular, and cranial assessments, this approach substantially enhanced management efficiency, reduced complications, and offered reliable technical support for the transport of high-risk neonates.

## 1 Introduction

The infant was a male born at 24 weeks’ gestation, had a maternal history positive for *Ureaplasma urealyticum* and *Candida glabrata* in vaginal secretions, incomplete antenatal dexamethasone for fetal lung maturation, and antepartum hemorrhage of approximately 500 mL, which necessitated urgent contact with the Dongguan Children's Hospital neonatal transport team. The infant was delivered vaginally with clear amniotic fluid and no nuchal cord and presented with shallow, weak breathing, cyanosis, poor response, low muscle tone, and a heart rate of 110 bpm. Initial management included warming, plastic wrap covering, endotracheal intubation, and T-piece positive-pressure ventilation. The Apgar scores were 6 at 1 and 5 min (1 point deducted each for color, respiration, tone, and reflex) and 7 at 10 min (1 point deducted each for respiration, tone, and reflex). The birth weight was 600 g. At 10 min postpartum, his respiratory distress worsened with the oxygen saturation (SpO₂) dropping to 65%–70%, necessitating intratracheal pulmonary surfactant (PS) instillation and initiation of ventilator support in the delivery room.

Upon arrival, the transport team assessed the patient's condition: (1) Under mechanical ventilation, the infant exhibited significant asynchrony and respiratory distress. The SpO₂ was 75%. Although the infant required an urgent lung assessment, bedside x-ray equipment was not immediately available. (2) To prevent hypoglycemia, establishing intravenous (IV) access for glucose infusion was critical. However, due to the fragile vasculature and coagulopathy of the extremely preterm infant, peripheral IV access posed a high risk of bleeding and bruising, making IV access extremely difficult. An urgent umbilical vein catheterization was planned with a low-positioned placement approach. (3) The difficulty in blood collection delayed the results of blood gas analysis. The transport team promptly utilized bedside ultrasound to evaluate and guide treatment strategies and obtained the following findings: (1) for the airway, a double-tract sign was visible on the transverse tracheal view, and the endotracheal tube was not displaced; (2) the lungs were present and showed symmetrical lung sliding bilaterally, absent A-lines, confluent B-lines, and sparse snowflake sign, symmetric diaphragmatic movement, and no pleural fluid; (3) in the vascular assessments, an umbilical venous catheter (UVC) was guided under ultrasound to the standard position above the diaphragm (low position unnecessary), and the umbilical arterial catheter (UAC) tip was guided to a position above the diaphragm at T6–T9 (lower half of the heart position); and (4) the cranial assessments showed no intracranial hemorrhage (ICH).

Comprehensive assessment confirmed correct and non-displaced endotracheal tube (ETT) position without over-insertion, and no pneumothorax (PTX) or pleural effusion. After PS therapy, lung ultrasound showed no significant pulmonary consolidation. On the basis of the lung-protective ventilation strategy for preterm infants, the ventilator pressure and inspiratory time were adjusted, and the more easily synchronized pressure control-assist control (PC-AC) mode was employed. After these adjustments, patient–ventilator synchrony was achieved, respiratory distress was alleviated, and oxygen saturation increased to 90%. Under ultrasound guidance, umbilical arterial and venous catheterization was successfully performed, establishing both arterial and venous vascular access. After initiating continuous intravenous glucose infusion, a blood gas sample was drawn from the UAC. The results were as follows: pH 7.211; partial pressure of carbon dioxide (pCO₂), 59.4 mmHg; partial pressure of oxygen (PO₂), 65 mmHg; bicarbonate level (HCO₃^−^), 23 mmol/L; base excess (BE), −4.6 mmol/L; glucose (GLU), 10.7 mmol/L; lactate (Lac), 8.68 mmol/L. On the basis of the results of blood gas analysis, further optimization of ventilator parameters and glucose infusion was continued. The infant was safely transferred to the neonatal intensive care unit (NICU) of Dongguan Children's Hospital under transport ventilator support, intravenous fluids, and warming in a transport incubator. Post-transfer, no further vascular access was needed; blood gas and glucose levels normalized; and intravenous nutrition continued. Confirmatory radiography verified correct positioning of the ETT, UAC, and UVC.

## 2 Discussion

Preterm infant transport is a core component of high-risk neonatal transport as well as a critical link in safeguarding infant health and improving outcomes. For fetuses with gestational age less than 33 weeks, *in utero* transfer to perinatal centers equipped with NICU capabilities is recommended (1). However, disparities in resource allocation and transport systems among primary care hospitals have resulted in low rates of implementation of *in utero* transfer. Consequently, a substantial proportion of very preterm infants fail to achieve *in utero* transfer. The transport process for extremely preterm infants (gestational age less than 28 weeks) and preterm infants with smaller gestational ages (less than 34 weeks) represents a high-risk phase for developing severe complications such as intraventricular hemorrhage (IVH), bronchopulmonary dysplasia (BPD), and patent ductus arteriosus (PDA) (2). Enhancing transport safety for this vulnerable population is currently a major focus in neonatal transport research. In this context, this report presents a case wherein POCUS was used to facilitate the safe interhospital transport of an extremely preterm infant with a gestational age of 24 weeks, providing empirical evidence to support technological innovations in high-risk preterm infant transport.

**Figure 1:**
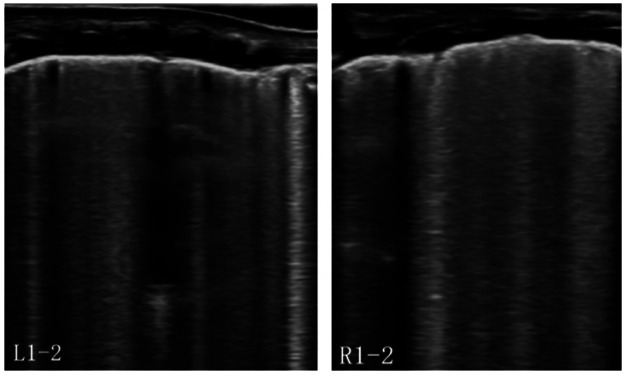
Lung ultrasound (LUS) revealed bilateral anterior pleural line irregularities, coalescent B-lines, and alveolar-interstitial syndrome (AIS). The A-lines were absent, ruling out PTX.

**Figure 2:**
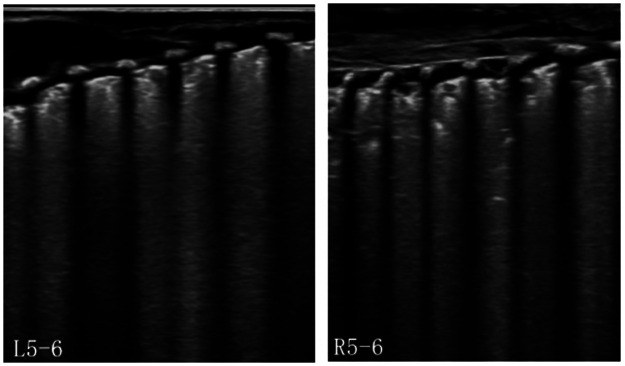
LUS showed discontinuous posterior pleural lines with small, scattered consolidations and a limited air bronchogram sign.

POCUS, which is characterized by non-invasiveness, real-time capability, and dynamic monitoring, is highly applicable for precise management of critically ill infants during transport, particularly when portable x-ray equipment is unavailable or cannot be promptly deployed. In clinical practice in Poland and Spain, LUS, a crucial component of POCUS, has demonstrated that the primary causes of respiratory failure in transported neonates include respiratory distress syndrome (RDS), transient tachypnea of the newborn (TTN), meconium aspiration syndrome (MAS), and PTX. The use of LUS both pre-transport and during transit can significantly improve respiratory management and stabilize patients (3, 4). This case involved an extremely preterm infant born at a gestational age of 24 weeks without receiving a full antenatal corticosteroid course for lung maturation. The infant exhibited persistent respiratory distress despite intratracheal PS administration and developed ventilator dyssynchrony and hypoxia, which required urgent lung evaluation. However, environmental constraints prevented timely access to portable x-ray equipment. Under these circumstances, LUS demonstrated critical advantages. It instantly excluded PTX by confirming lung sliding and B-line presence (Figure 1). After PS administration, decreased consolidation in both lungs indicated an improvement in alveolar collapse (Figure 2). The possibility of patient–ventilator asynchrony caused by suboptimal ventilator settings was considered. After adjusting the ventilation mode and inspiratory pressure, synchrony was achieved, and oxygen saturation stabilized/normalized. Thus, LUS enabled real-time assessment of therapeutic efficacy and played a critical role in guiding clinical decisions by monitoring pulmonary pathology and optimizing ventilator management.

**Figure 3:**
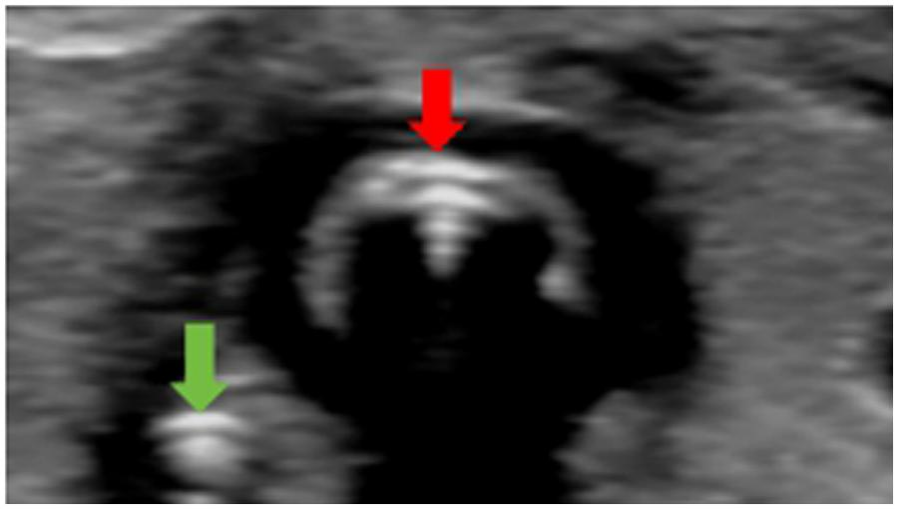
Using a linear probe for transverse scanning of the anterior neck, a double-track sign (indicated by the red arrow in the image) is visible in the transverse section of the trachea. The thyroid glands are located on both sides of the trachea. The green arrow in the lower left corner of the image indicates the position of the esophagus, which is adjacent to the trachea. Subsequently, a gastric tube was inserted, and the double-track sign was also observed under ultrasound.

In neonatal resuscitation scenarios involving a high risk of ETT displacement, traditional auscultation methods often struggle to provide accurate assessment of symmetric bilateral lung sounds due to the presence of environmental noise. Ultrasound assessments can enable real-time visualization of the tube position within the tracheal rings while concurrently showing bilateral lung sliding and the amplitude of diaphragmatic excursion. This allows for rapid confirmation of insertion depth and the exclusion of esophageal or bronchial misplacement, reducing the reliance on x-ray radiation exposure. A 2022 meta-analysis demonstrated that POCUS assessment of ETT position showed a sensitivity of 93.44% and a successful identification rate of 96.8%. This approach also significantly shortened the localization time in comparison with radiographic assessments, reducing it by over 30% on average. POCUS is easy to perform and suitable for emergency use in intensive care units and delivery rooms (5). In the present case, despite undergoing mechanical ventilation, the infant exhibited respiratory distress that necessitated assessment of tube position. The transverse tracheal view revealed the double-tract sign, a sonographic indicator of correct tube placement, confirming no tube displacement (Figure 3). Symmetric bilateral lung sliding and diaphragmatic excursion amplitude were observed, and the tube tip was positioned appropriately within the trachea. Subsequent chest radiography confirmed correct ETT positioning.

**Figure 4:**
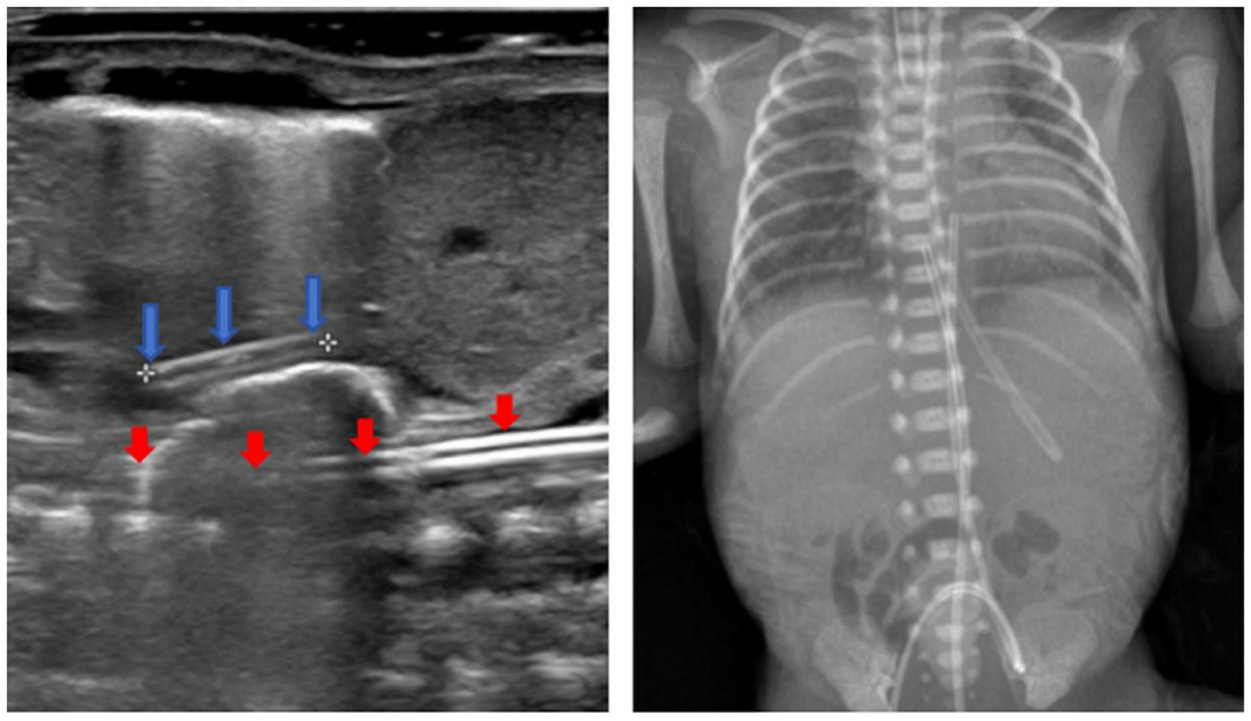
Point-of-care ultrasound (POCUS) confirmed the UVC tip approximately 1 cm above the diaphragm and and the UAC tip positioned at the mid-cardiac level. The corresponding radiograph demonstrated a UVC tip approximately 0.9 cm above the diaphragm and the UAC tip at the T7-T8 level. The endotracheal tube tip was positioned at the T3 vertebral level.The blue arrow in the image indicates the UVC, and the red arrow indicates the UAC.

Ensuring safe transport requires establishment of reliable IV access. However, peripheral IV access in extremely preterm infants is challenging, making umbilical venous catheterization the method of choice for fluid administration. Ultrasound guidance has significantly improved the safety and success rates of umbilical arterial and venous catheter tip placement in neonates. In comparison with the higher rates of complications (such as catheter malpositioning and vessel perforation) associated with traditional blind-placement techniques, ultrasound-guided real-time positioning can precisely locate the catheter tip, yielding success rates greater than 90% in positioning UVC tips at the ideal location (inferior vena cava–right atrial junction) (6). A randomized controlled trial conducted in 2025 demonstrated that the initial confirmation rate of correct positioning by radiography was significantly higher in the real-time ultrasound-assisted catheterization group than in the traditional formula-based method (*p* < 0.001), with the correct UAC placement rate also showing statistically significant improvement (*p* < 0.001). The ultrasound-guided umbilical vascular catheterization technique reduced the need for catheter repositioning and subsequent x-ray exposures (7). In such scenarios, establishment of venous access is critical to guarantee safe fluid administration, especially during transport of extremely preterm infants, whose fragile, small vessels pose substantial access challenges. In the present case, umbilical arterial and venous catheterization was successfully performed under ultrasound guidance in an extremely preterm infant, simultaneously addressing the difficulties associated with venous infusion and arterial blood sampling, thereby enabling secure transport conditions (Figure 4).

To mitigate the risk of ICH, serial cranial ultrasound monitoring before, during, and after transport should be performed for dynamic evaluation of extremely preterm infants. Close observation for the signs of ICH is essential throughout this process. This extremely preterm infant had a maternal history of antenatal bleeding, which represents a high-risk factor for ICH. However, cranial ultrasound assessments conducted before transport, upon arrival at our hospital, and within 72 h after birth revealed no signs of ICH. After 120 days of hospitalization, the infant clinically recovered and was discharged. Follow-up assessments until a corrected age of 18 months demonstrated normal physical growth and development. The Bayley Scales of Infant and Toddler Development assessment indicated moderate developmental levels in the intellectual and motor domains, reflecting a favorable prognosis with no neurological sequelae.

Thorough pre-transport preparation is essential for safe transport of extremely preterm infants. POCUS provides an essential foundation for formulating evidence-based transport plans by rapidly assessing key physiological parameters such as cardiac function; pulmonary, cerebral, and airway status; hemodynamics; and vascular access. This information can guide transport feasibility assessment, en route risk evaluation, and determination of resource requirements, including equipment and staffing (8, 9). By presenting this case study of transport for an extremely preterm infant, this report demonstrates how POCUS can enable non-invasive, immediate assessment of pulmonary pathology as well as precise localization of ETTs and UVCs, effectively replacing bedside x-ray examinations. These advantages can substantially optimize respiratory management and the establishment of reliable vascular access, thereby enhancing both transport safety and treatment outcomes for high-risk preterm infants. Nevertheless, whenever feasible, *in utero* transfer of the preterm fetus to a specialist center should be prioritized to secure the best possible outcome for both mother and infant.

The application of POCUS in pre-hospital critical care for high-risk neonates must be grounded in a standardized quality control system. In the present case, the transport team consisted of two associate chief neonatologists certified through the Chinese Critical Ultrasound Study Group (CCUSG) standardized training program, with both possessing systematic POCUS operational capabilities. A dual-review mechanism for image interpretation was implemented during resuscitation to ensure assessment accuracy, while a remote consultation channel was established to provide real-time support for complex cases. When the patient's condition permitted, complete ultrasound images were preserved as much as possible to facilitate subsequent case review and analysis. One limitation in this case was that the ultrasound examination time was minimized since the infant was an extremely preterm neonate with a gestational age of 24 weeks to reduce stimulation and avoid iatrogenic injury, resulting in some images not being saved. Furthermore, the widespread adoption of POCUS currently faces challenges such as limited device accessibility and operator proficiency. Future efforts should focus on promoting the application of POCUS in primary care institutions and pre-hospital transport scenarios, along with strengthening standardized operational training.

## Data Availability

The original contributions presented in the study are included in the article/Supplementary Material, further inquiries can be directed to the corresponding author.
